# The effect of bacterial mutation rate on the evolution of CRISPR-Cas adaptive immunity

**DOI:** 10.1098/rstb.2018.0094

**Published:** 2019-03-25

**Authors:** Anne Chevallereau, Sean Meaden, Stineke van Houte, Edze R. Westra, Clare Rollie

**Affiliations:** ESI and CEC, Biosciences, University of Exeter, Penryn Campus, Penryn, Cornwall TR10 9EZ, UK

**Keywords:** CRISPR-Cas adaptive immunity, bacteria, mutation rate, evolution, genetic variation, phage

## Abstract

CRISPR-Cas immune systems are present in around half of bacterial genomes. Given the specificity and adaptability of this immune mechanism, it is perhaps surprising that they are not more widespread. Recent insights into the requirement for specific host factors for the function of some CRISPR-Cas subtypes, as well as the negative epistasis between CRISPR-Cas and other host genes, have shed light on potential reasons for the partial distribution of this immune strategy in bacteria. In this study, we examined how mutations in the bacterial mismatch repair system, which are frequently observed in natural and clinical isolates and cause elevated host mutation rates, influence the evolution of CRISPR-Cas–mediated immunity. We found that hosts with a high mutation rate very rarely evolved CRISPR-based immunity to phage compared to wild-type hosts. We explored the reason for this effect and found that the higher frequency at which surface mutants pre-exist in the mutator host background causes them to rapidly become the dominant phenotype under phage infection. These findings suggest that natural variation in bacterial mutation rates may, therefore, influence the distribution of CRISPR-Cas adaptive immune systems.

This article is part of a discussion meeting issue ‘The ecology and evolution of prokaryotic CRISPR-Cas adaptive immune systems’.

## 1. Introduction

In the face of infection by bacteriophages (phage), bacteria have evolved a range of molecular mechanisms that provide immunity [1–4]. Arguably, one of their most sophisticated defences is CRISPR-Cas (Clustered Regularly Interspaced Short Palindromic Repeats; CRISPR-associated), an adaptive immune system. These immune systems are highly diverse and based on their *cas* gene synteny and CRISPR repeat sequences; they are currently classified into two classes, six types and 33 subtypes [5] that display clear differences in their molecular mechanisms of action. Nonetheless, all variants confer the ability to acquire sequence-specific phage resistance through the insertion of short pieces of phage-derived DNA (spacers) into CRISPR loci in the host genome (reviewed in [6]). Upon re-infection, processed transcripts of CRISPR loci (crRNA) guide CRISPR-associated (Cas) proteins to bind complementary sequences in the phage genome, followed by endonucleolytic cleavage of the phage DNA and/or RNA (depending on the CRISPR-Cas subtype) to clear the infection (reviewed in [7]).

Despite the obvious benefits of CRISPR-mediated phage resistance when phages are present in the environment [8], the majority of bacterial genomes lack a CRISPR-Cas adaptive immune system [9–13], an estimate that undoubtedly is subject to sampling biases, as some clades of unculturable bacteria appear to be essentially devoid of CRISPR systems [14]. This is in stark contrast with restriction-modification defence systems, which are present on average at two copies per cell [15] and raises the question why the fraction of bacterial genomes that encode CRISPR systems is so low. The common observation that CRISPR-Cas systems frequently move between species by horizontal gene transfer (HGT) suggests that the opportunity to acquire these systems is at least not a limiting factor, and instead suggests that these systems are frequently gained and subsequently lost again [12,16–24]. Several mutually non-exclusive explanations for this have been proposed. First, it appears to be the case that CRISPR-Cas systems are associated with autoimmunity issues due to self-targeting that could drive the loss of these systems from bacterial genomes [25–27]—a principle that is taken advantage of when applying CRISPR-Cas systems as antimicrobials [28–32]. In the context of lysogenization (i.e. when temperate phages integrate into the host genome), similar effects may occur, although at least some CRISPR-Cas variants appear to have mechanisms to provide some protection against self-cleavage in these instances [33,34]. An alternative explanation for the absence of CRISPR-Cas systems from many genomes is that they form a barrier for HGT. While this may in some cases be protective, it can also prevent the acquisition of potentially beneficial genetic information, and can, therefore, cause selection for bacteria with inactivated CRISPR-Cas systems when HGT is an important fitness determinant [27,35–37]. Yet, another explanation is that bacteria with CRISPR-Cas adaptive immunity can be outcompeted by bacteria with alternative defences under some ecological conditions [8,38–40]. Assuming there is a fitness trade-off associated with encoding CRISPR-Cas adaptive immune systems [41], then natural selection could favour loss of CRISPR-Cas systems in these environments (reviewed in [37,42]).

While each of these factors is likely to contribute to the overall phylogenetic distribution of CRISPR-Cas immune systems in bacteria, it is becoming increasingly clear that there are additional constraints that arise from the host genetic context. This is because the co-occurrence of certain non-*cas* genes is, in some cases, a prerequisite for encoding a fully functional CRISPR-Cas system, or because of epistatic interactions between non-*cas* genes and CRISPR-Cas systems. For example, during the first stage of the CRISPR-Cas immune response when new spacers are captured and integrated into the CRISPR array, the non-Cas protein, integration host factor (IHF), has been shown to be crucial in type I–E and I–F systems [43,44]. IHF guides spacer integration to the correct promoter-proximal end of the CRISPR array, where spacers provide the highest levels of resistance to re-infection [43–46]. Another example where an accessory host factor is essential for CRISPR functioning is in type II systems, which generally require RNase III for processing of the pre-CRISPR RNA transcript into short CRISPR RNA molecules (crRNA) that guide Cas complexes to target and destroy foreign elements [47–49].

Apart from these examples where host factors are essential, there are also instances of both positive and negative epistasis between CRISPR systems and other genes encoded by the same host. For example, type II-A CRISPR-Cas systems (specifically the Csn2 protein) inhibit the non-homologous end joining (NHEJ) DNA repair pathway, and as a consequence, these two systems almost never co-occur in the same genome [50]. The opposite can also happen, where systems act synergistically. For example, interference levels of *Escherichia coli* type I CRISPR-Cas systems depend on the presence of a homologue of heat shock protein 90 (HtpG) [51], the presence of a restriction-modification system has been shown to enhance the performance of a type II CRISPR-Cas immune system encoded by the same host [52], and it was reported that RecBCD-mediated DNA degradation products could feed into the spacer acquisition machinery of a type I CRISPR-Cas system [53].

While these examples illustrate how the host genetic context can determine whether or not acquisition of a CRISPR-Cas immune system would likely provide a fitness advantage, this list of examples is far from complete and our understanding of the way host genetic context and CRISPR-Cas interact is still rudimentary. Such knowledge is important for understanding the observed distribution of these systems, but also in an applied context, if we are to equip bacteria with CRISPR-Cas immunity to protect them against phage predation, for example, to protect fermentation in industrial settings.

Here, we examine whether bacterial mutation rates may form a barrier for the expression of the benefits associated with CRISPR-Cas adaptive immune systems. Bacterial mutation rates typically range from 1 in 10 million to 1 in a billion base substitutions per nucleotide per generation (reviewed in [54]), but bacteria with approximately 100-fold higher mutation frequencies are frequently found in both natural and clinical environments [55–57]. These high mutation rates are often due to inactivated mismatch repair systems and can either reduce or enhance bacterial fitness depending on the environment [58–61]. It is tempting to speculate that, in the context of phage predation, the benefits of CRISPR-Cas may be reduced in a host genetic background that has high mutation rates, because (i) these populations generate beneficial surface resistance mutations at a much higher frequency and (ii) high mutation rates may increase the rate at which CRISPR immunity is lost through mutation of *cas* genes or spacers in the CRISPR array [62].

To test these ideas, we performed experimental evolution with *Pseudomonas aeruginosa* strain UCBPP-PA14, a clinical isolate and model system for studying the evolution of CRISPR resistance in response to its phage DMS3*vir* [8,63]. *Pseudomonas aeruginosa* is an opportunistic pathogen that causes both acute and chronic infections in the lungs of cystic fibrosis (CF) patients, and its presence and the exacerbations it causes in CF lungs are generally considered to be one of the highest mortality risk factors for patients [64] (but also see [65] for a critical reflection on this dogma). Between one-third and one-half of CF patients with chronic *P. aeruginosa* infections harbour isolates with hypermutator phenotypes, typically due to mutations that inactivate the mismatch repair system, most commonly through mutation of the *mutS* gene [57,66,67]. By comparing the evolution of phage resistance in WT PA14 and isogenic *ΔmutS* strains, we find that high mutation rates have a dramatic impact on the evolution of phage resistance, which changes from being almost exclusively CRISPR-based in the WT background to being almost exclusively surface-based in the *ΔmutS* background. These data help us to understand how natural variation in mutation rates may impact the phylogenetic distribution of CRISPR-Cas systems, and have implications for phage therapy applications, where they may help to predict the relative importance of CRISPR- and surface-based resistances on the basis of the bacterial mutation rate.

## 2. Methods

### (a) Bacterial and virus strains

**Figure 1.**
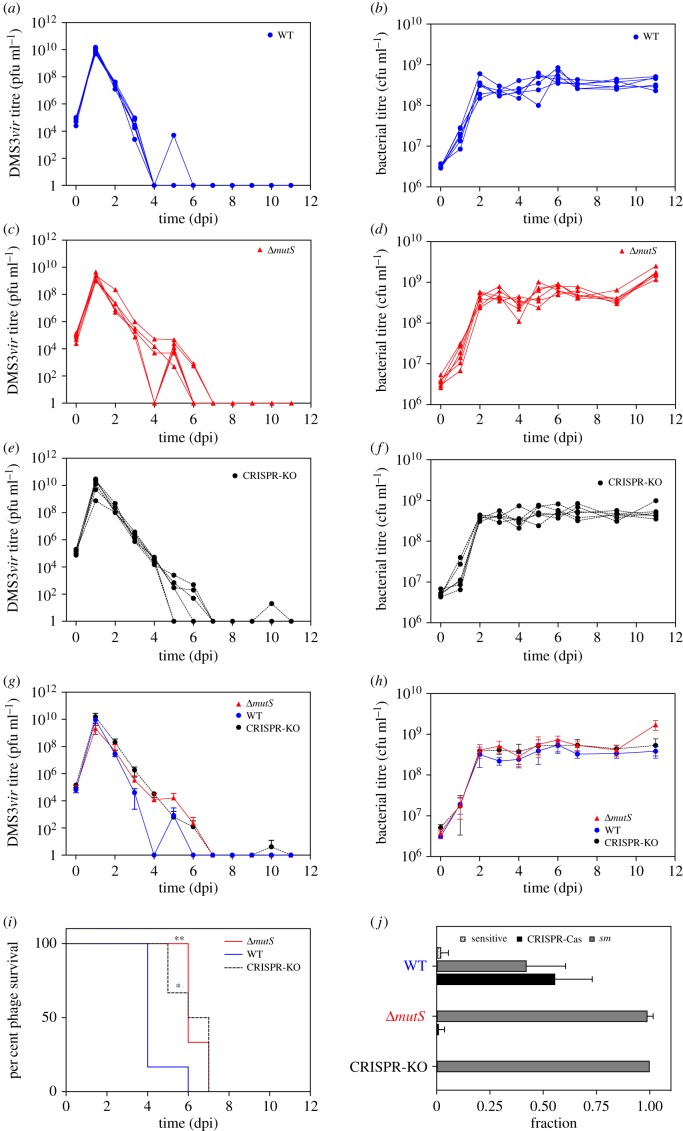
DMS3*vir* viral titre from 0 to 11 dpi of PA14 WT (*a*), PA14 Δ*mutS* (*c*) or PA14 CRISPR-KO (*e*) hosts. Bacterial titres during the course of the experiment were also measured for the same hosts (*b*), (*d*) and (*f*), respectively. The average (*N* = 6) phage (*g*) and bacterial (*h*) titres are displayed with error bars that represent 95% confidence intervals (CI). (*i*) Survival analysis of phage in different host backgrounds over the course of the experiment. (*j*) The immunity profile for each host at 3 dpi, showing the proportion of bacterial clones that evolved resistance by surface modification (*sm*) or CRISPR-Cas as well as those that did not evolve resistance (sensitive). Error bars represent 95% CI.

*Pseudomonas aeruginosa* UCBPP-PA14 (referred to as WT, carrying no spacers targeting DMS3*vir*), *P. aeruginosa* UCBPP-PA14 *csy3::LacZ* [63] (also referred to as CRISPR-KO, because it carries a disruption of an essential *cas* gene that causes the CRISPR-Cas system to be non-functional) and *P. aeruginosa* UCBPP-PA14 *mutS*::MAR2xT7 [68], which was kindly provided by Alexandro Rodriguez Rojas (below this strain is also referred to as *ΔmutS* or PA14 mutator strain), and the CRISPR-KO-derived surface mutant (*sm*) (described previously here [8]), were used in all experiments. WT or Δ*mutS* bacteriophage-insensitive mutants (BIM) (*N* = 6) isolated during the evolution experiment (figure 1), that had acquired two spacers against phage DMS3*vir,* were used in the competition experiment. Cells were grown overnight at 37°C in LB or M9 medium (22 mM Na_2_HPO_4_; 22 mM KH_2_PO_4_; 8.6 mM NaCl; 20 mM NH_4_Cl; 1 mM MgSO_4_; 0.1 mM CaCl_2_) supplemented with 0.2% glucose. The obligately lytic phage DMS3*vir* was used in all experiments, and has previously been described in [63]. DMS3*vir*-*acrIF1* was used in downstream analyses and has been described elsewhere [69]. Phage amplification and titrations were carried out on *P. aeruginosa* UCBPP-PA14 *csy3::LacZ*.

### (b) Evolution experiments

To monitor the evolution of bacterial resistance in response to phage infection and the associated bacterial and phage population dynamics, glass vials with 3 ml of LB medium or M9 medium supplemented with 0.2% glucose were inoculated with approximately 10^6^ bacteria from fresh overnight cultures of the corresponding bacterial strains. These cultures were infected with 10^4^ plaque forming units (pfu) of DMS3*vir*, followed by incubation at 37°C and shaking at 180 rpm. Cultures were transferred 1 : 100 into fresh medium every 24 h for 11 days (3 days only for LB). Experiments in M9 were performed in six independent replicates and those in LB in three independent replicates.

### (c) Measuring bacterial and phage population dynamics

Bacterial densities were determined by plating on LB agar serial dilutions of samples taken at each transfer in M9 salts (22 mM Na_2_HPO_4_; 22 mM KH_2_PO_4_; 8.6 mM NaCl; 20 mM NH_4_Cl; 1 mM MgSO_4_; 0.1 mM CaCl_2_). Phages were extracted at each transfer by chloroform extraction (sample : chloroform 10 : 1 v/v), and phage titres were determined by spotting serial dilutions of isolated phage samples in M9 salts on a lawn of CRISPR-KO bacteria.

### (d) Survival analyses

Phage survival analyses were carried out using GraphPad software by plotting the per cent survival phages at each time (Kaplan–Meier curve). Paired-comparisons of survival curves were made by applying the Mantel–Cox tests and were considered statistically significant when *p*-values were less than a Bonferroni-corrected threshold of 0.017.

### (e) Evolution of resistance

For consistency with previous studies [8,40,69,70], the evolution of resistance was determined at 3 days post-infection (dpi) by streaking individual colonies (always 16 randomly picked colonies per replicate) through DMS3*vir* and DMS3*vir*-*acrIF1*. Surface modification was confirmed by colony morphology, broad-range resistance to DMS3*vir* phages carrying *acr* genes, and lack of newly acquired spacers. CRISPR-Cas–mediated immunity was confirmed by PCR using primers 5′-CTAAGCCTTGTACGAAGTCTC-3′ and 5′-CGCCGAAGGCCAGCGCGCCGGTG-3′ for CRISPR array 1, and primers 5′-GCCGTCCAGAAGTCACCACCCG-3′ and 5′-TCAGCAAGTTACGAGACCTCG-3′ for CRISPR array 2.

### (f) Estimating frequency and rate of surface mutations in bacterial populations

Six colonies of WT, CRISPR-KO and Δ*mutS* strains were picked and cultured in 6 ml M9 medium overnight. These cultures were standardized to 0.1 OD_600_ and diluted 1000-fold. Fifty microlitres of each culture were used to seed 15 replicate populations per strain. After 24 h incubation (37°C, 180 RPM shaking), a 200 µl dilution series of each culture was exposed to 50 µl of either DMS3*vir* (approx. 10^10^ PFU ml^−1^, MOI approx. 500) or buffer and 5 µl immediately spotted on LB agar plates. The resulting drop plates were counted after 24 h incubation. The resistance phenotype of surviving colonies was confirmed by streaking colonies through DMS3*vir* and DMS3*vir-acrIF1*. Mutation rates were estimated from a Luria–Delbrück model using a maximum-likelihood method implemented by the *FLAN* package [71] in R (v. 3.5.1). Significance was determined using two-sample fluctuation analysis tests on mutant counts implemented using the flan.test function (*FLAN* [71]).

### (g) Competition assays to measure fitness

Competition experiments were performed in glass vials in 6 ml M9 medium supplemented with 0.2% glucose. Competition experiments were initiated by inoculating 1 : 100 from a 1 : 1 mixture of overnight cultures (grown in M9 medium + 0.2% glucose) of the CRISPR-KO-derived *sm* strain and either BIM of the WT or the Δ*mutS* strain with two spacers against DMS3*vir*, that had been isolated during the evolution experiment. Phage DMS3*vir* was added at the start of the experiment at 0, 10^4^, 10^7^ or 10^9^ pfu. Cells were transferred 1 : 100 daily into fresh broth. At 0, 1, 2 and 3 days, post-infection samples were taken and cells were serially diluted in M9 salts and plated on LB agar supplemented with 50 µg ml^−1^ X-gal (to allow discrimination between WT BIM or Δ*mutS* BIM bacteria (white) and *sm* (blue) bacteria). All experiments were performed in six replicates. Relative fitness was calculated from changes in the relative frequencies of blue and white colonies (rel. fitness = [(fraction strain A at *t* = *x*) * (1 − (fraction stain A at *t* = 0))]/[(fraction strain A at *t* = 0) * (1 − (fraction strain A at *t* = *x*)]).

## 3. Results

Clinical isolates of *P. aeruginosa* commonly have a hypermutator phenotype [57]. To understand if and how this impacts the benefits of a CRISPR-Cas adaptive immune system, we performed an evolution experiment and monitored the bacterial and phage population dynamics as well as the levels of CRISPR-mediated resistance that evolved following exposure of either WT PA14, a PA14 CRISPR-KO strain or a mutator (PA14 Δ*mutS*) strain to 10^4^ pfu of phage DMS3*vir* [63], a Mu-like phage [72] (figure 1*a–j*). *Pseudomonas aeruginosa* PA14 has a type I–F CRISPR-Cas system [11], which does not *a priori* target phage DMS3*vir* [63]. Consistent with previous studies [69], we found that following infection of the WT strain, phage titres rapidly increased, which is simply because bacteria are initially sensitive to phages and therefore allow rapid phage amplification. However, from 1 dpi onwards, phage titres started to decline rapidly until complete extinction at 6 dpi (figure 1*a*). As expected, bacterial densities remained low during the early stages of the experiment, but recovered from 2 dpi onwards, which coincided with the rapid decline in phage titres, and presumably reflects the evolution of phage resistance by the bacteria (figure 1*b*). In response to phage infection, *P. aeruginosa* strain UCBPP-PA14 can evolve either surface modification (*sm*) or CRISPR-Cas–mediated defence [8]. Analysis of individual bacterial clones that were isolated at 3 dpi revealed high levels of phage resistance evolution, and this was mainly due to CRISPR-mediated immunity of the bacteria (figure 1*j*).

Despite the previously reported benefit of high mutation rates to the bacteria when exposed to phage infection [60], when the same experiment was carried out using the PA14 Δ*mutS* strain, phage extinction risk was reduced compared to that observed for the WT strain (figure 1*c*,*g*,*i*, *p* = 0.005, Mantel–Cox test), and was similar to that observed following infection of the CRISPR-KO strain (figure 1*e*,*g*,*i*). Similar to what was observed for the WT strain, bacterial densities of the mutator and CRISPR-KO strains were initially low, but recovered from 2 dpi onwards, despite phage still being present (figure 1*d*). Interestingly, analysis of individual clones isolated at 3 dpi revealed that almost all PA14 Δ*mutS* bacteria had evolved surface-based resistance rather than CRISPR-based immunity (figure 1*j*). These findings help to explain why phage was able to persist for a longer period in the mutator background, because phages attempting to infect CRISPR-immune cells are destroyed, leading to a rapid reduction in their numbers. By contrast, phages cannot absorb to fully resistant surface-modified hosts and hence their numbers decrease more gradually through dilution by serial transfer. Additionally, a lack of phage extinction is commonly observed following evolution of surface-based resistance, probably because some surface modification mutants remain partially sensitive to phage infection [69]. To extend the generality of these findings, we also carried out this experiment in high nutrient LB media, which favours the evolution of surface-mediated resistance [8]. As expected, in these conditions, we found that WT bacteria evolved low levels of CRISPR-based resistance to DMS3*vir*, and mostly evolved resistance by surface modification. The CRISPR-KO and Δ*mutS* populations evolved resistance exclusively by surface modification in these conditions (electronic supplementary material, figure S1).

**Figure 2.**
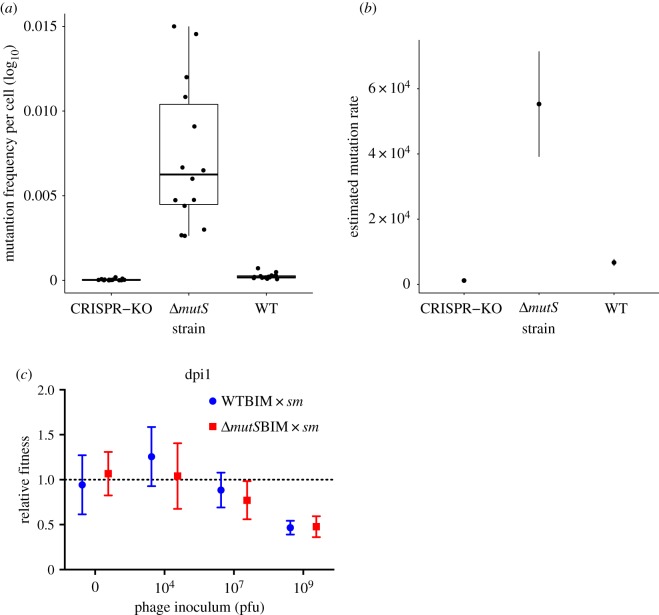
(*a*) Frequency of spontaneously generated surface mutants *(sm*) per cell for different PA14 hosts (*N* = 15). First and third quartiles are shown and whiskers represent 1.5× interquartile range. (*b*) Fluctuation test result calculated from data in (*a*) using a maximum-likelihood method showing estimated mutation rates of strains tested, error bars represent standard deviation (s.d.). (*c*) Relative fitness of BIM of WT or Δ*mutS* hosts that had evolved CRISPR-mediated resistance through the acquisition of two spacers against phage DMS3*vir*. These hosts were competed again a surface mutant (*sm*) in the presence of 0, 10^4^, 10^7^, 10^9^ pfus of phage DMS3*vir*. Data represent fitness at 1 dpi, *N* = 6, and error bars correspond to 95% CI.

While these data show that high bacterial mutation rates cause a strong reduction in the evolution of CRISPR resistance, it is unclear why this is the case. We envisaged two possible explanations (i) the frequency at which surface mutants are produced is higher in a mutator background and (ii) CRISPR-resistant clones in a mutator background are less fit than surface mutants, for example, because CRISPR immunity is rapidly lost as a consequence of mutations in the *cas* genes or CRISPR arrays. The first explanation is intuitively contributing to the observed effects, because bacterial clones evolve resistance in this system by random mutation of surface genes, particularly those that encode the pilus, which acts as the receptor for phage DMS3*vir* [8,63]. To formally test this hypothesis, we carried out a fluctuation test in which replicate populations of WT, Δ*mutS* and the CRISPR-KO were exposed to high phage titres before plating serial dilutions of the mixtures. By measuring the plated population size in the absence of phage and the number of survivors after phage exposure, we were able to calculate the frequency and rate of spontaneous surface mutation generation in each host background (figure 2*a,b*). The frequency of surface mutants was higher in the Δ*mutS* condition (mean of 7.34 × 10^−03^ per cell, 95% CI (4.89 × 10^−03^, 9.80 × 10^−03^)) compared to the CRISPR-KO (4.20 × 10^−05^, 95% CI (1.58 × 10^−05^, 6.82 × 10^−05^)) or WT background (2.37 × 10^−04^, 95% CI (1.45 × 10^−04^, 3.30 × 10^−04^)) (figure 2*a*). As the pilus locus is very large, comprising over 20 genes, the likelihood that it will acquire mutations is high and many of these will lead to a ‘surface mutant’ phenotype ([73]), which may explain the relatively high frequencies of phage-resistant surface mutants we see even in the WT and CRISPR-KO backgrounds. These data were entered into a modified form of the Luria–Delbrück mutation rate estimator that implements a maximum-likelihood method as described by Ycart & Veziris [74]. The calculated mutation rate of the Δ*mutS* strain to a surface mutant phenotype was significantly higher (mean = 5.53 × 10^−04^, s.d. = 1.62 × 10^−04^) than that of both the WT (6.71 × 10^−05^, s.d. = 1.09 × 10^−05^) and CRISPR-KO (1.17 × 10^−05^, s.d. = 2.74 × 10^−06^) hosts (two-sample ML test *t* = 4.69, *p* < 0.001, and *t* = 4.69, *p* < 0.001, respectively) (figure 2*b*). Surprisingly, we also found a small, but significant difference in the WT and CRISPR-KO mutation rates generated by this method (*t* = 3.9, *p* < 0.0001). Nonetheless, this analysis confirmed the prediction that the rate at which surface mutants are generated is higher (by approx. 10–50 fold) for the mutator background (figure 2*b*). To test the second explanation, we competed six independent CRISPR-resistant clones derived from the WT background or from the *ΔmutS* background against a previously described surface mutant (*sm*) that carries a *lacZ* marker gene [8], and which serves as a reference strain. The evolution of *sm* through loss of the phage receptor is frequently reported to be associated with a fixed fitness cost [8,75,76], whereas CRISPR-Cas–mediated defence is associated with an induced fitness cost [8]. Consistent with these previous observations, these competition experiments demonstrated that the relative fitness of CRISPR-resistant bacteria depends on the amount of phage (*p* ≤ 0.0001, *F*_3,40_ = 16.07, by two-way ANOVA), perhaps due to higher phage numbers leading to CRISPR-Cas being elicited more frequently and therefore to a higher inducible cost [8], but crucially, it is independent of the mutation rate of the host (*p* = 0.51, *F*_1,40_ = 0.44) (figure 2*c*). Collectively, these data therefore demonstrate that mutator strains evolve greater levels of surface resistance because these mutants pre-exist at higher frequencies in the population, and not because of a reduced selective advantage of CRISPR-resistant clones over surface mutants when the host has a higher mutation rate.

## 4. Discussion

It is commonly acknowledged that CRISPR-Cas adaptive immune systems frequently move by HGT and provide a benefit in the face of phage infection, yet they are found in less than half of the sequenced bacterial genomes. This apparent paradox may be partly explained by the loss of CRISPR-Cas systems due to immunopathological effects, i.e. the cytotoxic effects of self-targeting [25–27]. In addition, the system may form a barrier for HGT, driving its loss when HGT is an important fitness determinant [27,35]. Thirdly, other defences may be selected over CRISPR-Cas in some environments [8,38–40]. Finally, CRISPR-Cas systems may show negative epistasis with host genes, as was recently shown to be the case for the NHEJ DNA repair pathway and type II-A CRISPR-Cas systems [50]. Here, we show that mutation of the mismatch repair system, which results in a mutator phenotype, is associated with the virtually undetectable evolution of CRISPR-based resistance. We propose that the benefits of a CRISPR-Cas immune system are reduced in this genetic background, but this will need to be formally confirmed by performing competition experiments between Δ*mutS* strains with and without CRISPR-Cas in the presence/absence of phages. However, currently, we speculate that the natural variation in bacterial mutation rates may, therefore, influence the distribution of these adaptive immune systems.

Mutator phenotypes frequently arise both in the laboratory and in nature, most commonly due to frameshifts, insertions, premature stop codons or deletions of *mutS* or *mutL* genes [77,78]. These mutators have approximately a 100-fold increased rate of transition from G : C to A : T and vice versa, a 1000-fold increased rate of frameshift mutations, as well as a 10- to 1000-fold increase in the rate of chromosomal rearrangements [79]. Despite the increased rate at which these mutators accumulate deleterious mutations [58,80], they can sometimes outcompete non-mutators, particularly in fluctuating environments [58,81–86], such as those experienced during the antagonistic coevolution with phages [60]. The selective advantages of mutator phenotypes in fluctuating stressful environments also help to explain why mutators are relatively common in nature (sometimes with frequencies above 60%), and particularly so in pathogens (including, for example, *E. coli*, *Salmonella enterica*, *Neisseria meningitides*, *Haemophilus influenza*, *Staphylococcus aureus*, *Helicobacter pylori*, *Streptococcus pneumoniae* and *P. aeruginosa* (see [79] and references therein). Given that pathogens occupy niches where they are frequently exposed to various stressors, mutator phenotypes can provide a selective advantage by accelerating adaptive evolution [55,56,79]. For example, during colonization of the lungs of CF patients, *P. aeruginosa* strains are continuously exposed to osmotic and oxidative stress, the host immune system and antibiotics [64], and the observed appearance of mutator phenotypes during chronic infection may be important in driving rapid evolution of resistance to these factors [87,88]. This idea is further supported by the high frequency of mutator strains in chronically infected CF lungs (20% of isolates and 37% of patients carrying a mutator in one study [57]) and the positive relationship between mutator phenotypes and antibiotic resistance in pathogenic isolates of *P. aeruginosa* [57].

The relevance of mutator strains in clinical contexts, and the impact these phenotypes have on the evolution of phage resistance could be an important consideration in the context of phage therapy, which is currently undergoing a revival [89]. Our data show that—at least in an *in vitro* laboratory environment—mutator strains are much less likely to evolve CRISPR-based resistance and more likely to evolve surface resistance. Therefore, if the prevalence of mutator strains in an infection is known, it could be used to predict the relative importance of different resistance strategies likely to evolve during treatment. Furthermore, when CF patients carry mutator strains in their lungs, therapeutic use of phages may cause rapid emergence of surface mutants likely to outcompete other resistance strategies in the short term and become the dominant strain, which could have knock-on effects for the evolution of virulence and disease progression. We appreciate that the Δ*mutS* mutator strain chosen for these experiments is likely an extreme example, with a very high rate of mutations, and that a range of rates will exist in nature, with some mismatch repair mutants only having a slightly increased mutation supply rate compared to WT. This, in turn, may lead to variation in the importance of CRISPR-Cas in these hosts and further experiments will be needed to investigate this.

Apart from the biomedical implications, the results presented here help to shed further light on the factors that determine whether bacteria evolve surface resistance or CRISPR-based adaptive immunity against phages. Previous work with this same model system has shown that natural selection favours CRISPR-based defences if phage titres are low, because surface resistance is associated with a fixed cost of resistance, whereas CRISPR-based immunity is associated with a cost that is elicited only during phage infections [8]. Our data show that in addition to selection, the mutation supply rate also influences the type of phage resistance that evolves in this system. While we only tested the importance of mutation rates on the evolution of CRISPR-based versus surface-based resistance under laboratory conditions, if the same effects to apply in nature, it may, therefore, influence the benefits and hence the distribution of CRISPR-Cas systems. Future studies are needed to examine whether such correlations exist between CRISPR activity and host mutation rates.

## Supplementary Material

Supplemental Figure 1

## Data accessibility

Statistical analyses were carried out using the GraphPad Prism 7 software and R (v. 3.5.1). Raw data files from the experiments are available from the Dryad Digital Repository: http://dx.doi.org/10.5061/dryad.937s037 [90].
