# Supplementary material for: The effect of bacterial mutation rate on the evolution of CRISPR-Cas adaptive immunity
Source: Philos Trans R Soc Lond B Biol Sci. 2019 Mar 25;374(1772):20180094. doi: 10.1098/rstb.2018.0094 (PMC6452272; doi:10.1098/rstb.2018.0094)
Supplement: Supplemental Figure 1 [file rstb20180094supp1.docx]

Supplemental Figure 1. The immunity profile for each host after a 3 day coevolution experiment with DMS3*vir* in nutrient rich LB medium, showing the proportion of bacterial clones that evolved resistance by surface modification (*sm*) or CRISPR-Cas as well as those that did not evolve resistance (Sensitive). Error bars represent 95 % CI.
